# Trastuzumab resistance induces EMT to transform HER2^+^ PTEN^−^ to a triple negative breast cancer that requires unique treatment options

**DOI:** 10.1038/srep15821

**Published:** 2015-11-02

**Authors:** Joseph P. Burnett, Hasan Korkaya, Maria D. Ouzounova, Hui Jiang, Sarah J. Conley, Bryan W. Newman, Lichao Sun, Jamie N. Connarn, Ching-Shih Chen, Ning Zhang, Max S. Wicha, Duxin Sun

**Affiliations:** 1 Department of Pharmaceutical Sciences, The University of Michigan, 428 Church St., Ann Arbor, MI 48109-1065; 2 Department of Biochemistry and Molecular Biology, Georgia Regents University Cancer Center, 1410 Laney Walker Boulevard CN2136 Augusta, GA 30912; 3 Department of Biostatistics, The University of Michigan, 1415 Washington Heights, Ann Arbor, MI 48109-2029; 4 Department of Internal Medicine, University of Michigan Comprehensive Cancer Center, 1500 East Medical Center Drive, Ann Arbor, MI 48109-5942; 5 Division of Medicinal Chemistry, College of Pharmacy, The Ohio State University, 50 W 12th Ave, Columbus, OH 43210; 6 Tianjin Key Laboratory on Technologies Enabling Development of Clinical Therapeutics and Diagnostics (Theranostics), Research Center of Basic Medical Science & Cancer Institute and Hospital, Tianjin Medical University, No. 22 Qixiangtai Road, Heping District, Tianjin 300070, People’s Republic of China

## Abstract

Although trastuzumab is an effective treatment in early stage HER2^+^ breast cancer the majority of advanced HER2^+^ breast cancers develop trastuzumab resistance, especially in the 40% of breast cancers with loss of PTEN. However, HER2^+^ breast cancer patients continue to receive trastuzumab regardless PTEN status and the consequence of therapy in these patients is unknown. We demonstrate that continued use of trastuzumab in HER2^+^ cells with loss of PTEN induces the epithelial-mesenchymal transition (EMT) and transform HER2^+^ to a triple negative breast cancer. These transformed cells exhibited mesenchymal morphology and gene expression markers, while parent HER2^+^ cells showed epithelial morphology and markers. The transformed cells exhibited loss of dependence on ERBB family signaling (such as HER2, HER3, HER4, BTC, HRG, EGF) and reduced estrogen and progesterone receptors. Continued use of trastuzumab in HER2^+^ PTEN^−^ cells increased the frequency of cancer stem cells (CSCs) and metastasis potential. Strikingly, parental HER2^+^ cells and transformed resistant cells respond to treatment differently. Transformed resistant cells were sensitive to chemical probe (sulforaphane) through inhibition of IL-6/STAT3/NF-κB positive feedback loop whereas parental HER2^+^ cells did not respond. This data suggests that trastuzumab resistance in HER2^+^ PTEN_−_ breast cancer induces EMT and subtype switching, which requires unique treatment options.

The development of anti-HER2 targeted therapy (trastuzumab) has significantly improved the survival of HER2^+^ breast cancer patients. However, initial response rates in women with HER2 overexpressing metastatic disease treated with single agent trastuzumab range from only 11.6–34%^1^,^2^. Further, the majority of patients given trastuzumab treatment will develop drug resistance within one to two years^3^,^4^. Therefore, it is necessary to identify potential mechanisms of trastuzumab resistance and develop alternative therapeutics for trastuzumab-resistant HER2^+^ breast cancers.

Previous studies have revealed compensatory signaling mechanisms responsible for the drug resistance of HER2^+^ breast cancer, including: inactivation of PTEN tumor suppressor; antigen masking on HER2 epitope by MUC4; enhanced signaling through other ERBB family receptors; cross-talk of HER2 with IGF-1R; and mutational activation of downstream signaling through PI3-K/AKT pathway^5^,^6^,^7^,^8^,^9^. Inactivation of the PTEN tumor suppressor, found in ~40% of patients with HER2 overexpression, has been demonstrated to induce drug resistance in tumor xenografts and correlate with trastuzumab resistance in patients^10^. In addition, inactivation of PTEN has been shown to be a crucial factor inducing epithelial to mesenchymal transition (EMT) in breast, colon, nasopharyngeal, and prostate cancers^11^,^12^,^13^,^14^. Furthermore, recent evidence suggests that EMT may activate diverse alternative survival pathways or actually transform the molecular subtype of the malignancy in castration/enzalutamide resistant prostate cancer, RAF inhibitor resistant melanoma, and EGFR inhibitor resistant lung cancer^15^,^16^,^17^,^18^.

Korkaya *et al.* previously reported that drug resistance in HER2 overexpressing cell lines with PTEN deletion by long term culture with trastuzumab (LTT) induces characteristics of the EMT and expands the breast cancer stem cell (BCSC) population^19^. This induction of EMT and expansion of cancer stem cells is proposed to occur through activation of an IL-6/NF-κB positive feedback loop. Interestingly, several studies have demonstrated that inflammatory cytokines such as IL-6 are upregulated in triple negative breast cancers (TNBC) and correlated with poor patient prognosis^20^,^21^,^22^,^23^.

Regardless of PTEN status, HER2^+^ patients continue to be given trastuzumab in current clinical practice. However, the clinical consequence for the continued use of trastuzumab in HER2^+^ PTEN^−^ breast cancer is unknown, especially after resistance is developed. In this study, we report that continued use of trastuzumab to induce resistance in PTEN deficient HER2^+^ breast cancer results in the epithelial to mesenchymal transition (EMT), as evident by reduced expression of epithelial markers and increased mesenchymal makers. Following EMT, trastuzumab resistant PTEN deficient breast cancer cells transform HER2^+^ cells to a more aggressive TNBC phenotype with reduction in HER2, estrogen, and progesterone receptor expression while increasing proliferation. Furthermore, these transformed trastuzumab resistant cells exhibit increased BCSC populations and rate of metastasis. Strikingly, the parental HER2^+^ cells and transformed resistant cells respond to treatment uniquely, where transformed resistant cells were sensitive to chemical probe (sulforaphane) through inhibition of IL-6/STAT3/NF-κB positive feedback loop and parental HER2^+^ cells failed to respond to sulforaphane. Sulforaphane selectively eliminated both BCSCs and bulk PTEN deficient, trastuzumab-resistant, breast cancer proliferation. These results suggest that continued use of trastuzumab in HER2^+^ breast cancer with loss of PTEN function induces EMT, and consequent transforms molecular subtype from HER2^+^ to triple negative, which requires unique treatment options to improve patient survival.

## Results

### Continued use of trastuzumab in HER2^+^ breast cancer with PTEN inactivation generates resistance and induces characteristics of the epithelial to mesenchymal transition (EMT)

**Figure 1:**
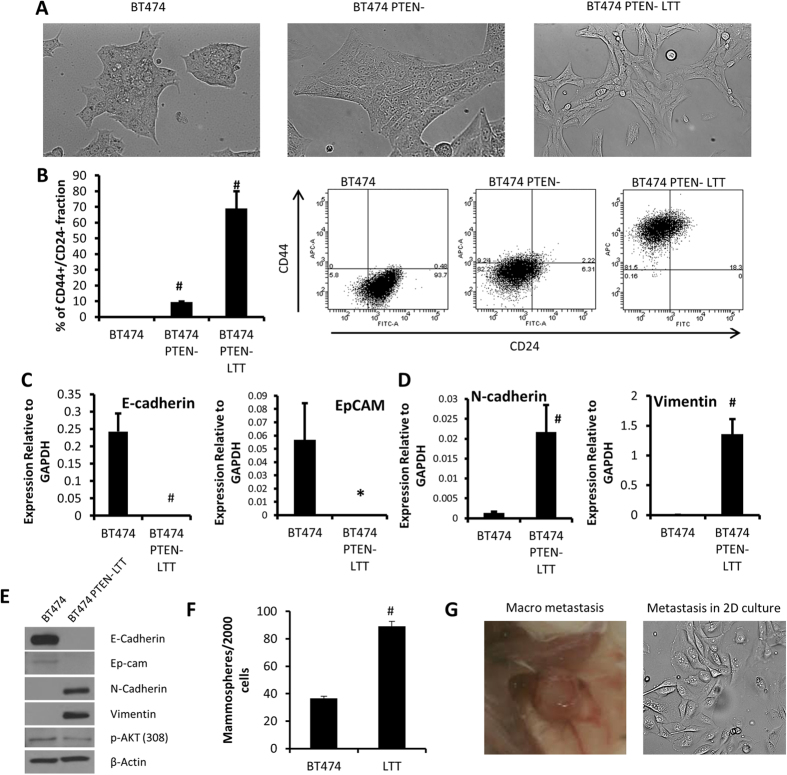
Continued use of trastuzumab in HER2^+^ breast cancer with PTEN inactivation generates resistance, induces a mesenchymal phenotype, and increases cancer stem cells. (**A**) Representative bright field microscopy images of BT474, BT474 shPTEN, and BT474 shPTEN with long term treatment of trastuzumab (LTT). Original images obtained with 20× objective. (**B**) Left, percent of cells expressing basal breast cancer markers CD44+/CD24- in cell lines as assessed by flow cytometry. N = 3. Right, representative CD44 and CD24 flow cytometry analysis of each. (**C**) Real time PCR quantification of epithelial cell makers E-cadherin and EpCAM normalized to expression of GAPDH. (**D**) Real time PCR analysis of mesenchymal cell markers N-cadherin and Vimentin, expressed relative to GAPDH. N = 4. (**E**) Western blot analysis of protein level expression of EMT related genes. (**F**) Mammosphere formation rates of parental BT474 and BT474 PTEN^−^ LTT cell lines. N = 3. (**G**) Representative image of macrometastasis that forms in 1 in 5 mice BT474 PTEN^−^ LTT xenograft bearing mice before primary tumors reach 500 mm3. Data shown as average ± SD. *p ≤ 0.05, #p ≤ 0.01.

In HER2 amplified breast cancer cell line BT474, lentiviral vector containing shPTEN was used to knockdown PTEN as reported previously^19^. This cell line (BT474 PTEN^−^) was cultured long-term with trastuzumab (LTT, >3 weeks) to induce stable trastuzumab resistance (BT474 PTEN^−^ LTT). In order to establish morphological and phenotypic properties of trastuzumab-sensitive and resistant cell lines, we analyzed these cells utilizing microscopy and flow cytometry. Bright field microscopy reveals each sequential step in the generation of trastuzumab resistance results in adaption of a more mesenchymal like phenotype (Fig. 1A). In addition, there was a dramatic increase in expression of basal like markers CD44+ CD24− in trastuzumab resistant BT474 PTEN^−^ LTT cells (69.1%) as well as PTEN deleted cells (9.5%) compared to parental BT474 which lacks this population (Fig. 1B).

To further characterize the parental and trastuzumab resistant cell lines, real time PCR analysis of EMT related genes was performed. While parental BT474 exhibit high expression of both E-cadherin and EpCAM, these epithelial markers are at undetectable levels in the BT474 PTEN^−^ LTT cell line (Fig. 1C). Conversely, the expression of mesenchymal cell markers N-cadherin and vimentin are increased by 16 and 266-fold respectively (Fig. 1D). To confirm the protein level changes Western blot analysis of these epithelial and mesenchymal makers was performed. Similarly, N-cadherin and vimentin were detected in BT474 PTEN^−^ LTT cells, but not in parental BT474 cells. In contrast, E-cadherin and EpCAM were detected in parental BT474 cells, but not in BT474 PTEN^−^ LTT cells (Fig. 1E). This data confirms the continued use of trastuzumab generates resistance in HER2^+^ PTEN deficient breast cancer cells and induces characteristics of the EMT.

### Continued use of trastuzumab in HER2^+^ breast cancer with PTEN inactivation expands cancer stem cells and increases metastatic potential *in vivo*

In order to validate the reports of Korkaya *et al.*, that expansion of breast CSCs follows the generation of trastuzumab resistance, mammosphere formation assay was performed^24^. Only 1.83% of cells from parental BT474 were capable of mammosphere formation whereas BT474 PTEN^−^ LTT cells exhibited a 2.4-fold higher formation rate (Fig. 1F). When implanted as orthotopic mouse xenografts trastuzumab resistant BT474 PTEN^−^ LTT cells were capable of producing macro-metastasis in lymph nodes, which could be subcultured *in vitro*, whereas parental BT474 xenografts were not (Fig. 1G). Macrometastasis were identified in one in five mice before primary tumors reached 500 mm^3^. Further, when mice bearing BT474 PTEN LTT xenografts were treated with trastuzumab the frequency of tumor initiating cells was increased 4.7 fold in secondary reimplantation assay (discussed below). Together these results suggest that continued use of trastuzumab in PTEN deficient HER2^+^ breast cancer results in EMT and expansion of breast CSCs.

### Continued use of trastuzumab in HER2^+^ breast cancer with PTEN inactivation induces subtype switching from HER2^+^ to a triple negative like breast cancer

**Figure 2:**
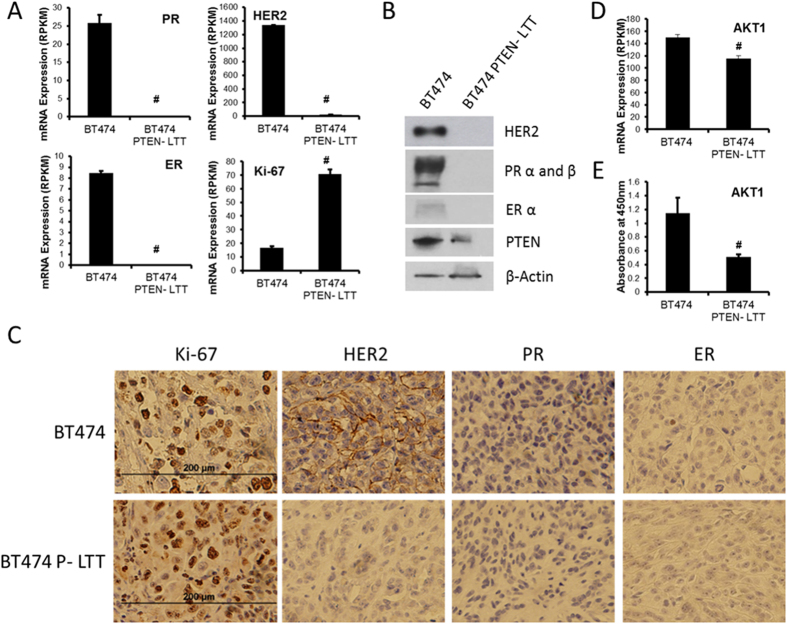
Continued use of trastuzumab in HER2^+^ breast cancer with PTEN inactivation induces subtype switching from HER2^+^ to a triple negative like breast cancer. (**A**) Normalized mRNA expression (reads/kilobase/million reads) of breast cancer subtype markers ER, PR, HER2, and Ki-67 determined by RNA sequencing in Parental BT474 and BT474 PTEN^−^ LTT cell lines N = 4.(**B**) Western blot representing breast cancer subtype markers ER, PR, HER2 and PTEN with β-actin as loading control. (**C**) Immunohistological staining of breast cancer subtype markers in primary BT474 and BT474 PTEN^−^ LTT xenografts when tumors reached 500 mm^3^. (**D**) mRNA expression of HER2 signal transducer AKT1 as determined by RNA sequencing. (**E**) Normalized protein expression of AKT1 in cell lines by ELISA assay. N = 3. Data shown as average ± SD. #p ≤ 0.01.

In order to explore the potential changes in diverse signaling pathways which may be differentially regulated following the induction of EMT by trastuzumab we utilized RNA sequencing. Strikingly, the expression of classical breast cancer subtype markers ER and PR were reduced to near undetectable levels while HER2 was reduced 64-fold and Ki-67 mRNA was increased 4-fold (Fig. 2A). In addition to the mRNA reduction of classical breast cancer subtype markers, expression of ERBB receptor family members HER3 and HER4, as well as the major ligands associated with all major ERBB family signaling molecules (EGF, BTC, HRG) were reduced (Supplemental Fig. 1A). Further, genes previously reported to be associated with trastuzumab resistance such as MUC4 and IGF-1R show only modest changes in their expression (Supplemental Fig. 1B). Consistent with mRNA levels determined by RNA-seq the protein level of ER, PR, and HER2 were significantly reduced as demonstrated by western blot analysis (Fig. 2B). When grown in an orthotopic mouse xenograft model BT474 cells exhibit strong HER2 staining, intermediate Ki-67, low PR, and no ER as determined by immunohistochemistry. In contrast, BT474 PTEN^−^ LTT xenografts *in vivo* exhibit a higher Ki-67 expression and low to undetectable levels of HER2, PR and ER (Fig. 2C).

ERBB family members are characterized by their homo and hetero dimerization upon ligand binding and capable of activation of PI3K and downstream AKT signaling nodes. Consistent with a reduction in HER2 following induction of trastuzumab resistance, RNA sequencing revealed a 23% reduction in AKT1 mRNA (Fig. 2D). Similarly, relative quantitation of AKT1 protein expression was reduced 2.25-fold, as observed by enzyme-linked immunosorbent assay (ELISA), in BT474 PTEN^−^ LTT cells when compared to the parental cell line (Fig. 2E). In addition, western blot analysis shows a reduction in the activating phospho-Thr308 post translational modification (Fig. 1E). These data suggest that continued use trastuzumab generates resistance and subtype switching from HER2^+^ breast cancer to a triple negative subtype breast cancer.

### Continued use of trastuzumab in HER2^+^ breast cancer with PTEN inactivation switches signaling pathway dependence from HER/AKT to IL-6/STAT3/NF-κB signaling

**Figure 3:**
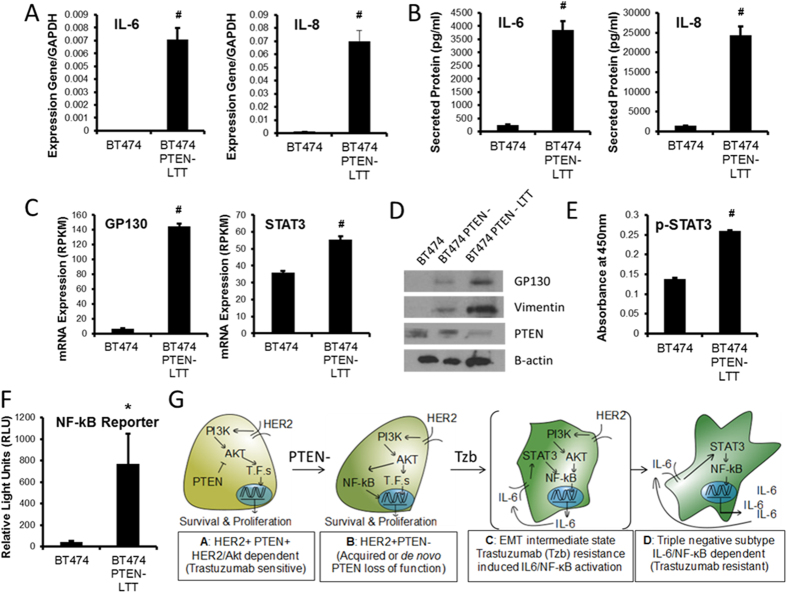
Continued use of trastuzumab in HER2^+^ breast cancer with PTEN inactivation switches signaling pathway dependence from HER/AKT to IL-6/STAT3/NF-κB signaling. (**A**) mRNA expression of endogenous inflammatory cytokines IL-6 and IL-8 normalized to GAPDH as identified by real time PCR. N = 4 (**B**) Protein expression in pg/ml of IL-6 and IL-8 secreted into culture media, measured using quantitative enzyme-linked immunosorbent assay (ELISA). N = 3 (**C**) Normalized mRNA expression (reads/kilobase/million reads) of IL-6 signal transducer GP130 (left) and downstream signaling node STAT3 (right) by RNA-sequencing. N = 4 (**D**) Western blot analysis of protein expression for GP130, mesenchymal cell marker vimentin, and PTEN tumor suppressor in BT474, BT474 PTEN^−^, and BT474 PTEN^−^ LTT cell lines. (**E**) ELISA relative quantitation of active STAT3, phosphorylated at Tyr705. N = 3 (**F**) Luciferase activity of cell lines following infection of with retroviral vector inducing NF-κB driven expression of luciferase. N = 3. (**G**) Model of trastuzumab resistance in PTEN deficient cells, T.F.s = other transcription factors. Data shown as average ± SD. *p ≤ 0.05. #p ≤ 0.01.

The observation that classical breast cancer subtype markers are down regulated suggests another signal transduction pathway may play a critical role in proliferation of BT474 PTEN^−^ LTT cells. Recent evidence from several groups suggest that triple negative breast cancers exhibit a preferential expression of inflammatory cytokines such as IL-6 and IL-8^23^,^25^. Consistent with previous studies^19^, real time PCR demonstrated >50-fold up regulation of IL-6 and IL-8 mRNA following the generation of trastuzumab resistance (Fig. 3A). A quantitative ELISA showed that IL-6 and IL-8 secreted into cell culture media after 72 hr exhibited a 15 and 17-fold enhancement respectively when compared to parental cell line (BT474) (Fig. 3B).

Iliopoulos and colleagues propose that IL-6 activation of STAT3, through binding to its receptors IL6R and complex formation with GP130, elicits NF-κB mediated IL-6 production, thus generating a positive feedback loop capable of transforming immortalized breast cell line MCF10A^26^. In later work this group and others identified that microRNA regulation of PTEN and CYLD is a critical link in this “epigenetic switch”, and that IL-6 production in PTEN deficient cells is capable of inducing EMT^27^. RNA sequencing results demonstrate that expression of IL-6 co-receptor GP130, and downstream signaling node STAT3 were increased 22 fold and 54% respectively in BT474 PTEN^−^ LTT cells (Fig. 3C). Enhanced production of GP130 mRNA was consistent with the stepwise increase in protein as the parental cell line BT474 was subjected to PTEN deletion and long term culture with trastuzumab (Fig. 3D, vimentin increase is displayed as indicator of the EMT). Further, increased production of IL-6 and GP130 was accompanied by an 86.7% increase in p-STAT3 as determined by ELISA (Fig. 3E). Since STAT3 has also been shown to activate NF-κB to promote IL-6 production^27^, a luciferase reporter driven by the NF-κB transcriptional response element (TRE) was employed and demonstrated that BT474 PTEN^−^ LTT cells exhibit 17.5 -fold higher NF-κB activity relative to parental BT474 (Fig. 3F).

In order to evaluate if the observed increased activity of NF-κB and STAT3 represent dependence for cell viability and CSC characteristics, siRNA knockdown of NF-κB and STAT3 was performed in BT474 PTEN^−^ LTT cells. Between 24 and 48 hours post transfection, knockdown of NF-κB reduced viability 38.8% whereas knockdown of STAT3 reduced the number of total viable cells by 14.3% (Supplemental Fig. 2A). The siRNA knockdown of NF-κB and STAT3 appear to induce necrotic cell death (by DAPI staining), as there was no evidence of differences in early apoptosis (Annexin V+ DAPI- staining) between siRNA knockdown of NF-κB, STAT3, and siRNA scrambled control (data not shown). Further, following knockdown of both NF-κB and STAT3, mammosphere formation was reduced by 29.3% and 51.5, respectively, in BT474 PTEN^−^ LTT cells (Supplemental Fig. 2B). Together these results suggest that trastuzumab resistance in PTEN deficient HER2^+^ breast cancer results in the EMT to transforms PTEN deficient HER2^+^ breast cancer to a triple negative phenotype that is dependent upon the IL-6/STAT3/NF-κB signaling axis for survival and self-renewal of CSCs (Fig. 3G).

### Parental HER2^+^ and transformed resistant cells respond uniquely to therapy *in vitro*

**Figure 4:**
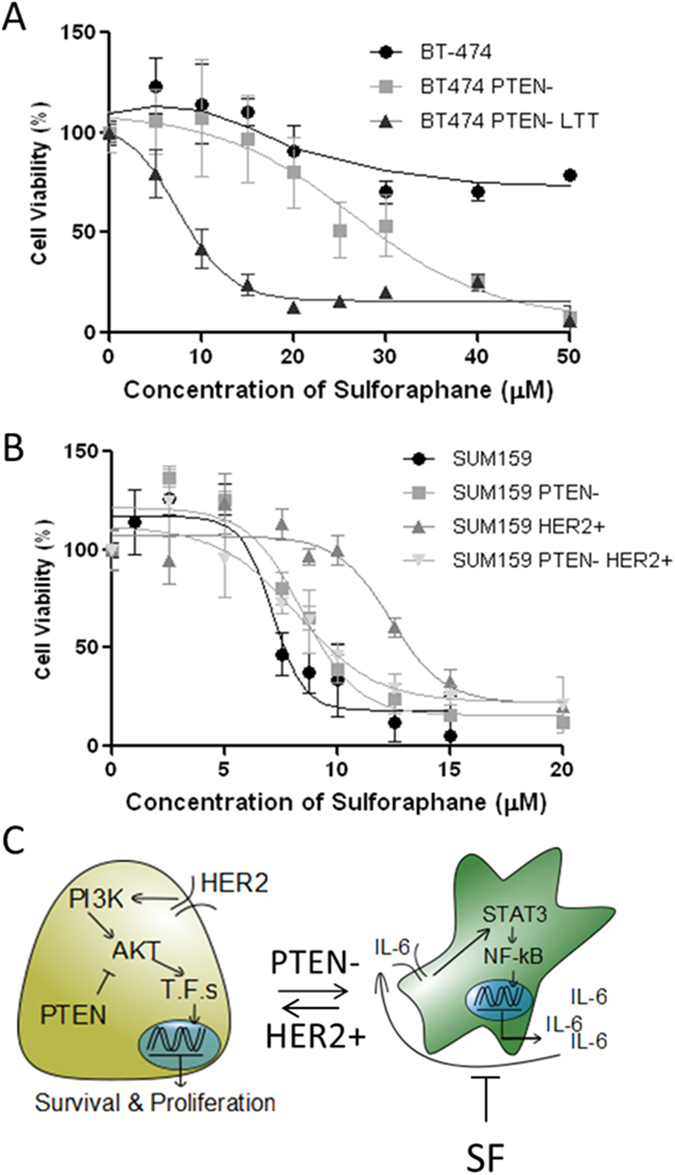
Parental HER2^+^ and transformed resistant cells respond uniquely to therapy *in vitro*. MTS proliferation assay (Promega) was carried out after incubating cell lines with increasing concentration of SF for 72 hours. (**A**) PTEN knock down and long term trastuzumab (LTT) culture with HER2 amplified BT474 sensitizes the cell line to SF. (**B**) MTS assay performed in triple negative SUM159, SUM 159 with shPTEN, SUM159 with HER2 over expression, and SUM159 shPTEN with HER2 overexpression. (**C**) Model of SF efficacy demonstrating cell lines with HER2 amplification in the absence of PTEN deletion are likely to be insensitive to SF treatment whereas deletion of PTEN is likely to enhance efficacy. (**A**,**B**) N = 6. Data are shown as averages ± SD.

EMT and subtype switching, along with signaling pathway transitions, in trastuzumab resistance breast cancer cells with PTEN inactivation suggest that these transformed cells may require unique treatment options. Functional activation of canonical NF-κB signaling requires phosphorylation of IκB and subsequent nuclear translocation of NF-κB subunits from the cytoplasm followed by DNA binding and initiation of transcription^28^. Previous studies have shown that a small chemical probe, sulforaphane (SF), was able to inhibit nuclear translocation of p65 as assessed by western blot in human prostate cancer cell line PC-3 and breast cancer cell line MCF-7^29^,^30^. Thus, we utilized the MTS proliferation assay to compare sensitivity of BT474, BT474 PTEN^−^, and BT474 PTEN^−^ LTT cells to SF (Fig. 4A). SF exhibited a poor reduction in proliferation of BT474 at concentrations as high as 50 μM over 72 hours. Strikingly, deletion of PTEN resulted in a sensitization of the cell line to SF. BT474 PTEN^−^ LTT cells further displayed a marked increase in their sensitivity to SF by 5-fold.

Previous reports from our laboratory have identified the efficacy of SF in claudin-low SUM159 is similar to these transformed PTEN deficient breast cancer cells^31^, which is significantly different when compared to that of HER2 amplified BT474. To identify if the presence of HER2 is responsible for reduced efficacy of SF, we overexpressed this receptor in SUM159. Indeed, overexpression of HER2 in SUM159 cells resulted in a near doubling of IC50 from 7.63 to 13.22 μM (Fig. 4B). Furthermore, shRNA knockdown of PTEN in SUM159 HER2^+^ cells, restored sensitivity of these cells to sulforaphane. Taken together, these results suggest that SF preferentially suppresses proliferation in cell lines which primarily rely on IL-6/NF-κB for their survival, whereas strong activation of HER2/AKT signaling may predict resistance to SF (Fig. 4C).

### Sensitivity of breast cancer stem cells and bulk tumor volume to therapy is unique between parental HER2^+^ and transformed resistant cells *in vivo*

**Figure 5:**
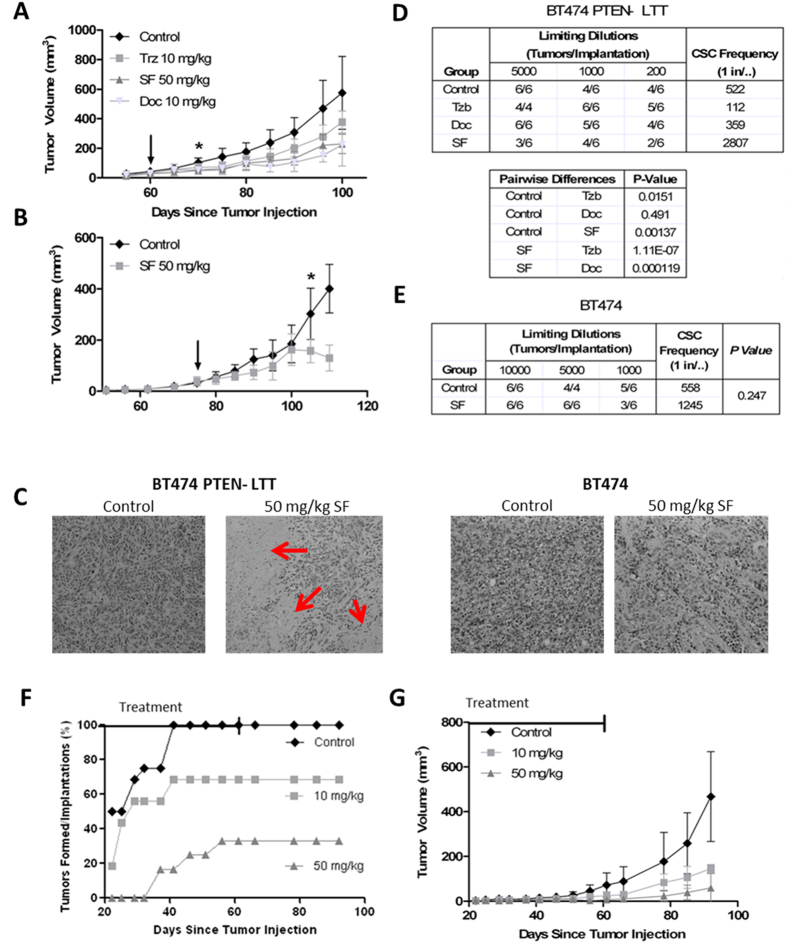
Sensitivity of breast cancer stem cells and bulk tumor volume to therapy is unique between parental HER2^+^ and transformed resistant cells *in vivo*. (**A**) NOD/SCID mice bearing 40 mm^3^ BT474 PTEN^−^ LTT xenografts were randomized into treatment groups, 50 mg/kg SF and 10 mg/kg Doc were sufficient to significantly reduce bulk tumor volume within 10 days. Trastuzumab treatment produced no significant reduction in tumor volume for BT474 PTEN^−^ LTT xenografts over the course of the experiment. *p ≤ 0.05. (**B**) Tumor volume of mice bearing primary BT474 xenografts, 30 doses of SF were required to significantly reduce tumor volume. Arrow denotes the beginning of treatment in primary mice. *p ≤ 0.05. (**A,B**) Tumor volume represented SD of N = 6 mice. (**C**) Representative H&E staining of primary BT47 and BT474 PTEN^−^ LTT tumors. SF treatment in BT474 PTEN^−^ LTT xenografts generated intratumoral regions of necrosis (red arrows) whereas this was not observed in BT474 xenografts. Primary xenografts were removed, dissociated, and serially reimplanted into secondary mice to determine the tumor initiating CSC frequency by extreme limiting dilution analysis of primary (**D**) BT474 PTEN^−^ LTT and (**E**) BT474 xenografts. (**F**) Tumor formation rate of mice injected with 50,000 BT474 PTEN^−^ LTT cells followed 2 days later by daily SF treatment. Control N = 16, 10 mg/kg N = 16, 50 mg/kg N = 12. (**F**) Average volume of BT474 PTEN^−^ LTT xenografts that formed with adjuvant treatment. Control N = 16, 10 mg/kg N = 9, 50 mg/kg N = 4.

To identify that the parental HER2^+^ cells and transformed trastuzumab resistant cells respond to treatment uniquely with respect to conventional (docetaxel and trastuzumab) and experimental therapies (sulforaphane) an advanced treatment orthotopic mouse xenograft model was employed. BT474 PTEN^−^ LTT and BT474 cells were implanted into NOD/SCID mice and treatment began after the tumor volume reached 40 mm^3^. Docetaxel and trastuzumab were administered via IP injection once weekly while sulforaphane (SF) was administered daily. In BT474 PTEN^−^ LTT xenograft bearing mice, 10 doses of SF significantly reduced tumor volume by 51.8% (Fig. 5A). Daily SF treatment ultimately resulted in a 61.0% reduction in tumor volume relative to saline control, an effect that is comparable to that of docetaxel (60.1%) which serves as a positive control for bulk tumor volume reduction. As expected, treatment of mice with weekly IP injections of trastuzumab resulted in no statistically significant reduction in tumor volume. Conversely, in BT474 xenografts SF was only able to produce a significant reduction in tumor volume (47.4%) after 30 doses (Fig. 5B). Histological staining of primary xenografts using haematoxylin and eosin reveal dramatically larger areas of necrosis with BT474 PTEN^−^ LTT tumors treated with SF in comparison with SF treated BT474 or control xenografts (Fig. 5C).

To identify if SF preferentially reduced CSCs (Fig. 5D,E) in BT474 PTEN^−^ LTT versus parental BT474 tumors secondary reimplantation assay with extreme limiting dilution analysis (ELDA) was employed^32^. When control tumor volumes reached protocol specific endpoint xenografts were dissociated into a single cell suspensions and FACS sorted for the use in secondary reimplantation assays. Nine weeks post-reimplantation of vehicle treated tumors from mice bearing BT474 PTEN^−^ LTT xenografts demonstrated a tumor initiating frequency of 1/522 cells (Fig. 5D). In contrast, residual tumors from sulforaphane (SF) treated mice showed significantly lower tumor initiating frequency (1/2807 cells) when implanted into secondary mouse fat pads. BT474 PTEN^−^ LTT xenografts treated with trastuzumab had a significant increase in frequency of tumor initiating cells (1/112 cells p = 0.015 vs. 1/522 cells in control group). Docetaxel treatment in mice failed to eliminate the breast CSC population in tumors, consistent with previous reports^33^. Secondary implantation from BT474 xenografts treated with SF demonstrated only a modest decrease in tumor initiating cell frequency, with 1/1245 cells in SF treated group vs.1/558 cells in control group p = 0.247 (Fig. 5D).

In order to evaluate if transformed trastuzumab resistant cells would respond to SF treatment as adjuvant therapy in patients following surgical resection of tumors, and inhibit breast CSC tumor initiation, we utilized an early treatment xenograft model. Two days following inoculation of BT474 PTEN^−^ LTT cells, animals were randomized and treated with 10 mg/kg SF, 50 mg/kg SF, or saline vehicle. Daily dosing was continued until control tumors reached a volume of 100 mm^3^ at which point treatment was stopped and tumor monitoring was continued. Over the course of treatment 50 mg/kg SF prevented 66.7% of tumors from forming in mice implanted with BT474 PTEN^−^ LTT, an effect persistent 30 days after discontinuation of treatment (Fig. 5F). Furthermore, administration of 10 mg/kg reduced tumor formation 31.25%, revealing a dose dependent therapeutic effect. In tumors which did form, SF treatment reduced bulk volume by 68.7% and 87.3% at doses 10 and 50 mg/kg respectively (Fig. 5G).

### Sensitivity of transformed trastuzumab resistant PTEN^−^ breast cancer to sulforaphane is mediated by inhibition of IL-6/NF-κB signaling loop

**Figure 6:**
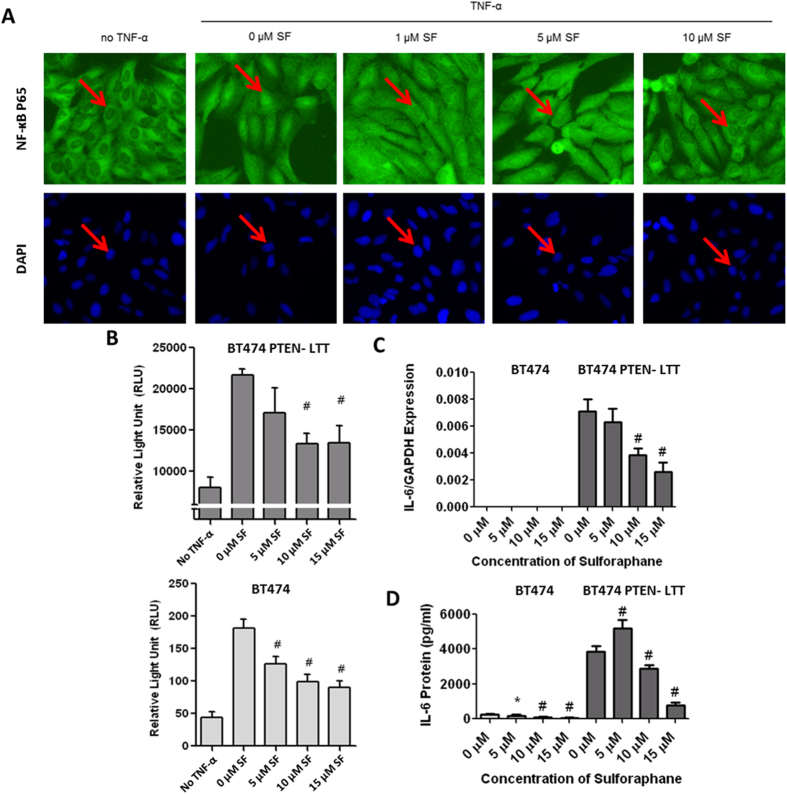
Sensitivity of transformed trastuzumab resistant PTEN^−^ breast cancer to sulforaphane is mediated by inhibition of IL-6/NF-κB signaling loop. (**A**) Immunofluorescent staining of NF-κB p65 subunit and DAPI in fixed BT474 PTEN^−^ LTT cells exhibits primarily cytoplasmic staining (left). Translocation of p65 from cytoplasm to nucleus, induced using 50 ng/ml TNF-α, is prevented by pretreatment with SF in a dose dependent manner (right three panels). Red arrows indicate boarder between cytoplasm and nucleus of a representative cell. Original images were obtained using a 20× microscope objective. (**B**) BT474 PTEN^−^ LTT and BT474 cell lines were transfected with NF-κB luciferase reporter. Luciferase reporter activity was obtained in the absence or presence of 50 ng/ml TNF-α in combination with increasing concentrations of SF. (**C)** mRNA expression of endogenous inflammatory cytokine IL-6 normalized to GAPDH as identified by real time PCR in the presence of increasing concentration of SF. (**D**) Quantitative ELISA identifying protein level endogenous NF-κB target protein IL-6 when cell lines are incubated with increasing concentrations of SF for 72 hr. (**B–D**) N = 3. Data are shown as averages ± SD. *p ≤ 0.05. #p ≤ 0.01.

We sought to determine if the sensitivity of the transformed, trastuzumab resistant, PTEN inactivated breast cancer to SF was related NF-κB nuclear translocation. Under typical culture conditions p65 resides primarily in the cytoplasm of BT474 PTEN^−^ LTT cells. Following the addition of tumor necrosis factor alpha (TNF-α) in the absence of SF, the NF-κB p65 subunit translocated to the nucleus, as evident by the disappearance of a clearly defined nuclear border (Fig. 6A, two left panels). However, stimulation of cells with TNF-α in the presence of increasing concentration of SF blocked p65 NF-κB nuclear translocation in a dose dependent manner, with inhibition occurring in some cells at concentrations as low as 1 μM (Fig. 6A, right panels). Similar results were observed in other breast cancer cell lines (luminal B type MCF7 and triple negative SUM159 cells).

In order to elucidate whether the inhibition of NF-κB intracellular translocation by SF also reduces its transcriptional activity, we employed the NF-κB luciferase reporter assay. Following stimulation of BT474 or BT474 PTEN^−^ LLT cell lines with TNF-α, luciferase activity increases 4.14 and 2.70-fold (Fig. 6B). The enhanced reporter activity is substantially reduced by 61% in a dose dependent manner by SF treatment in both BT474 and BT474 PTEN^−^ LTT. Similar results were also observed in other triple negative breast cancer cells (SUM159 and MDA-MB-231).

To assess the ability of SF to further regulate endogenous NF-κB targets, we employed real time PCR and ELISA to monitor the mRNA and protein levels of IL-6. We found SF reduced both mRNA level and secretion of IL-6 by more than 70% in both BT474 and BT474 PTEN^−^ LTT cell lines (Fig. 6C,D). Together, these findings provide evidence that sulforaphane (SF) is able to disrupt the IL-6/NF-κB positive feedback loop that PTEN inactivated, trastuzumab resistant, breast cancers are reliant on.

## Discussion

Contribution of the PTEN tumor suppressor to resistance against trastuzumab in HER2 amplified breast cancer patients has been previously reported in the literature. Inactivation of PTEN is associated with reduced function of trastuzumab in BT474 and SKBR3 cell lines *in vitro* and correlates with poor response to trastuzumab in patients^10^,^34^. However the translation of these findings to clinical application of trastuzumab to patients has yet to be fully realized, where HER2 amplified patients regardless of PTEN status are usually treated with trastuzumab in combination with surgery, radiotherapy, and conventional chemotherapies depending upon expression of other hormone receptors, tumor size, lymph node status, and the presence of distant metastasis. Since it is unknown what the potential clinical consequences of continued use of trastuzumab as a therapy in HER+ PTEN deficient patients we sought to explore this using genetic manipulation and drug conditioning in the breast cancer cell line BT474.

Consistent with a previous report we demonstrate that long term culture with trastuzumab in BT474 containing reduced PTEN function (shPTEN) induces morphological and transcriptional changes characteristic of the epithelial to mesenchymal transition in a stepwise manner^19^. These results suggest deletion of PTEN may be critical for priming the cells for further transformation. Iliopoulos *et al.* have demonstrated that microRNA targeting of PTEN is a critical step in triggering transformation of the immortalized MCF-10A cell line by IL-6^26^,^27^. More recently, transformation of MCF-10A by PTEN and p53 knockdown has been shown to generate a triple negative type breast cancer cell line^25^.

Upon further characterization of BT474 PTEN^−^ LTT cells, we identified that expression of classical breast cancer cell markers ER, PR, and HER2 were all significantly reduced at mRNA and protein level both *in vitro* and *in vivo*, while expression of proliferation marker Ki-67 was increased. This stably resistant cell also exhibited a reduced mRNA and protein level of downstream signaling node AKT1 which can be regulated by multiple ERBB family members. This is consistent with previous reports demonstrating that culture with trastuzumab significantly down regulates HER2 protein in the HER2^+^ MDA-MB-453, which contains mutations in both PIK3CA and PTEN^35^,^36^,^37^. Multiple studies have utilized BT474 in xenograft model, treated with trastuzumab, which provide insight into the molecular mechanisms associated with acquired trastuzumab resistance^38^,^39^. *In vivo*, these studies report trastuzumab treatment does not cause a significant down regulation of HER2 and resistant cell lines remain dependent on ERBB family signaling. However, our data showed that continued use of trastuzumab in HER2^+^ breast cancer with PTEN inactivation induces EMT and results in the conversion to a triple negative molecular subtype. Taken together with our results these studies may suggest that the generation of acquired and *de novo* trastuzumab resistance exhibit two distinct molecular mechanisms, with patients exhibiting PTEN deletion at high risk for the observed molecular subtype switching.

Following the generation of trastuzumab resistance in HER2^+^ breast cancer cells with PTEN deletion, the mRNA and protein level of inflammatory cytokines IL-6 and IL-8 were dramatically unregulated. Indeed we postulate that IL-6 is the main driver of the EMT induction process. Constitutive expression of IL-6 in MCF-7 results in a dramatic reduction in E-cadherin while up regulating mesenchymal markers N-cadherin, vimentin, and twist^40^. Similarly, IL-6 stimulation of CD44-CD24+ breast cancer patient derived cells has been demonstrated to reduce E-cadherin expression and generate a CD44 + CD24- population. Further, the induction of EMT in Ras-transformed mammary epithelial cells has also been shown to rely on NF-κB signaling^41^, which has previously been shown to be activated through the IL-6/STAT3/NF-κB positive feedback loop^13^,^17^,^26^. Consistent with these reports we find that with the reduction of HER2/AKT signaling there is a significant increase in IL-6, GP130, level of p-STAT3, and activity of the NF-κB transcription factor.

Strikingly, parental HER2^+^ BT474 and transformed drug resistant HER2^+^ PTEN deficient cells showed dramatically difference in response to chemical probe (SF) treatment. In previous reports SF has been demonstrated to inhibit NF-κB transcriptional activity by preventing p65 DNA binding activity in pancreatic and breast cancer cell lines^30^,^42^. Additionally, in prostate cancer cell lines SF reduced NF-κB nuclear translocation and transcriptional activity^43^. Further, SF’s activity in pancreatic CSCs has been attributed to inhibition of the NF-κB transcription factor^42^,^44^. In this report, we demonstrate that SF is capable of disrupting NF-κB p65 translocation, transcriptional activity, and inhibiting endogenous target IL-6 at mRNA and secreted protein level across breast cancer cell lines. While parental BT474 did not respond to SF treatment, BT474 with PTEN deletion and long term culture with trastuzumab showed more than 5-fold sensitivity than BT474 both *in vitro* and in mouse xenograft model. Additionally, the inhibitory effect of SF on both tumor volume and tumor initiation in secondary mice was preferentially observed in trastuzumab-resistant BT474 PTEN^−^ LTT. Of note, the reduction of bulk tumor volume by SF in trastuzumab-resistant xenografts, at the indicated dosing regimen, was comparable to that of conventional chemotherapy while the additional ability to reduce tumor initiating cell frequency was observed.

These data suggest that continued use of trasuzumab in HER2^+^ breast cancer patients with PTEN inactivation may pose potential challenges for second line targeted therapies due to induction of EMT, expansion of BCSC populations, and by switching the molecular subtype to triple negative. Additional clinical investigation may be warranted to explore the role of this phenomenon in breast cancer patients with different status of PTEN and HER2. Based on these findings novel therapeutic options need to be explored for the treatment of trastuzumab resistant breast cancers with PTEN inactivation.

## Materials and Methods

### Cell Culture and Reagents

BT474 was cultured in DMEM supplemented with 10% fetal bovine serum and 1% antibiotic-antimycotic under a 5% CO2 environment. BT474^−^ PTEN^−^ LTT cells, which were induced by shRNA knockdown of PTEN and long term treatment of trastuzumab, were maintained in the same media as the parental cell line. Sulforaphane was obtained from Quality Phytochemicals LLC (New Jersey, USA) and diluted in DMSO (<0.1%) for *in vitro* studies or 0.9% saline *in vivo*. Docetaxel (Hospira) and Trastuzumab (Herceptin, Genentech) were obtained through the University of Michigan Cancer Center Pharmacy.

### Quantification of mRNA

Total RNA from cell lines was extracted using RNeasy mini kit (Qiagen) according to manufacturer’s protocol. Purified total RNA was further prepared for RNA-sequencing using TruSeq RNA sample preparation kit (Illumina) followed by 50 cycle, single end reads carried out using the HiSeq 2000 sequencing system (Illumina). RNA expression analysis was performed by normalization using reads per kilobase per million reads (RPKM) method. RNA purified for real time PCR analysis was converted to cDNA using the M-MLV RT (Promega) and subjected to real time PCR analysis using TaqMan universal PCR master mix (Roche) and indicated primers using ABI PRISM 7900GT sequencing detection system (Applied Biosystems).

### Luciferase Reporter Assay

Lentiviral particles containing luciferase reporter construct driven by NF-κB (System Biosciences) were obtained and transfected into BT474 and BT474 PTEN^−^ LTT cells. Two hours following incubation with SF, TNF-α concentration in media was brought to 50 ng/ml. After 6 hr Luciferase activity was measured according to manufacturer’s instructions with oneGlo reagent (promega) on Synergy 2 plate reader (BioTek).

### Protein Expression Analysis

After culture for 72 hours media or cells were collected and subjected to ELISA for Human IL-6, IL-8, AKT, and p-STAT3. Assays were performed using antibody kits for AKT and p-STAT3 (Cell Signaling Technology) or IL-6 and IL-8 (Duosets, R&D Systems) according to manufacturer’s protocol. Data was acquired with a BioTek Synergy plate reader and analyzed using Gen5 software. Antibodies for western blot (HER2, PR α/β, ER α, PTEN, GP130, Vimentin, β-actin, and secondary antibodies) were obtained from Cell Signaling Technology.

### Flow Cytometry and Immunostaining

Flow cytometry analysis of cell lines *in vitro* was performed using anti-CD44-APC (Cat.#559942), anti-CD24-FITC (Cat.# 555427), and corresponding isotype antibodies (BD Biosciences) on a SY3200 (Sony Biotechnology) flow cytometer with a minimum of 10,000 live cells detected. Immunofluorescent staining of p65 NF-κB (Cell Signaling Technology) in BT474 PTEN^−^ LTT cell line was performed in 4-well glass chambers slides (lab-tek) following pretreatment with SF for 2 hours and 30 min and 50 ng/ml TNF-α for 2 hours. Cells were fixed and permeabilized with methanol/acetone followed by blocking with 3% BSA. Nuclear staining was identified with 1 μg/ml DAPI and imaging was carried out with a Nikon Eclipse TE2000-S microscope and MetaMorph 7.6.0.0 (Molecular Devices).

### MTS Cell Proliferation Assay

Cell lines were plated at a density of 3,000 cells per well in 96-well plates and allowed to adhere overnight. Following 72 hour incubation with SF proliferation was determined by MTS assay according to manufacturer’s instruction. Absorbance at 490 nm was measured using Synergy 2 plate reader. Pharmacodynamic modeling was performed using a nonlinear, variable slope model in GraphPad Prism (GraphPad Software).

### Mouse Xenograft Models

All experimental studies involving the use of vertebrates were carried out in accordance with the U.S. Government Principles for the Utilization and Care of Vertebrate Animals Used in Testing, Research, and Training. The following experiments were approved by the University Committee on the Use and Care of Animals at the University of Michigan. Female 5 week old non-obese diabetic/severe combined immunodeficient (NOD/SCID) mice were obtained from Jackson Laboratory. Xenograft formation in advanced treatment model was generated by direct injection of BT474 or BT474 PTEN^−^ LTT cells (1,000,000), suspended in matrigel (BD Biosciences), into the exposed no.4 inguinal mammary pad. Trastuzumab (10 mg/kg once every 7 days), sulforaphane (50 mg/kg daily), and docetaxel (10 mg/kg once every 7 days) were administered via I.P. injection beginning when tumor volume reached approximately 40 mm^3^ or the day after surgery for adjuvant treatment model. When control tumors reached approximately 500 mm^3^ mice were euthanized with CO2 inhalation and tumors resected.

### Secondary Reimplantation Assay

Isolated primary tumors were mechanically and enzymatically dissociated using gentleMACS Octo Dissociator and tumor dissociation kit (Miltenyi Biotec) according to manufacturer’s instructions. Human tumor cells with DsRed label were then isolated using fluorescent activated cell sorting on a SY3200 (Sony Biotechnology) flow cytometer. Secondary mice were inoculated with 10,000, 5,000, or 1,000 cells for BT474 xenografts and 5,000, 1,000, or 200 cells for BT474 PTEN^−^ LTT xenografts as described above. Tumor formation rate in secondary mice was assessed 9 weeks following implanting cells by direct palpitation.

## Additional Information

**How to cite this article**: Burnett, J. P. *et al.* Trastuzumab resistance induces EMT to transform HER2^+^ PTEN^−^ to a triple negative breast cancer that requires unique treatment options. *Sci. Rep.* **5**, 15821; doi: 10.1038/srep15821 (2015).

## Supplementary Material

Supplementary Information
