# Supplementary material for: Trastuzumab resistance induces EMT to transform HER2+ PTEN− to a triple negative breast cancer that requires unique treatment options
Source: Sci Rep. 2015 Nov 2;5:15821. doi: 10.1038/srep15821 (PMC4629137; doi:10.1038/srep15821)
Supplement: Supplementary Information [file srep15821-s1.pdf]

Trastuzumab resistance induces EMT to transform $HER2^{+}PTEN^{-}$ to a triple negative breast cancer that requires unique treatment options

Joseph P. Burnett[1], Hasan Korkaya[2], Maria D. Ouzounova[2], Hui Jiang[3], Sarah J. Conley[4], Bryan W. Newman[1], Lichao Sun[1], Jamie N. Connarn[1], Ching-Shih Chen[5], Ning Zhang[6], Max S. Wicha[4], Duxin Sun[1*].

[1] Department of Pharmaceutical Sciences, The University of Michigan, 428 Church St., Ann Arbor, MI 48109-1065
[2] Department of Biochemistry and Molecular Biology, Georgia Regents University Cancer Center, 1410 Laney Walker Boulevard CN2136 Augusta, GA 30912
[3] Department of Biostatistics, The University of Michigan, 1415 Washington Heights, Ann Arbor, MI 48109-2029
[4] Department of Internal Medicine, University of Michigan Comprehensive Cancer Center, 1500 East Medical Center Drive, Ann Arbor, MI 48109-5942
[5] Division of Medicinal Chemistry, College of Pharmacy, The Ohio State University, 50 W 12th Ave, Columbus, OH 43210
[6] Tianjin Key Laboratory on Technologies Enabling Development of Clinical Therapeutics and Diagnostics (Theranostics), Research Center of Basic Medical Science & Cancer Institute and Hospital, Tianjin Medical University, No. 22 Qixiangtai Road, Heping District, Tianjin 300070, People's Republic of China

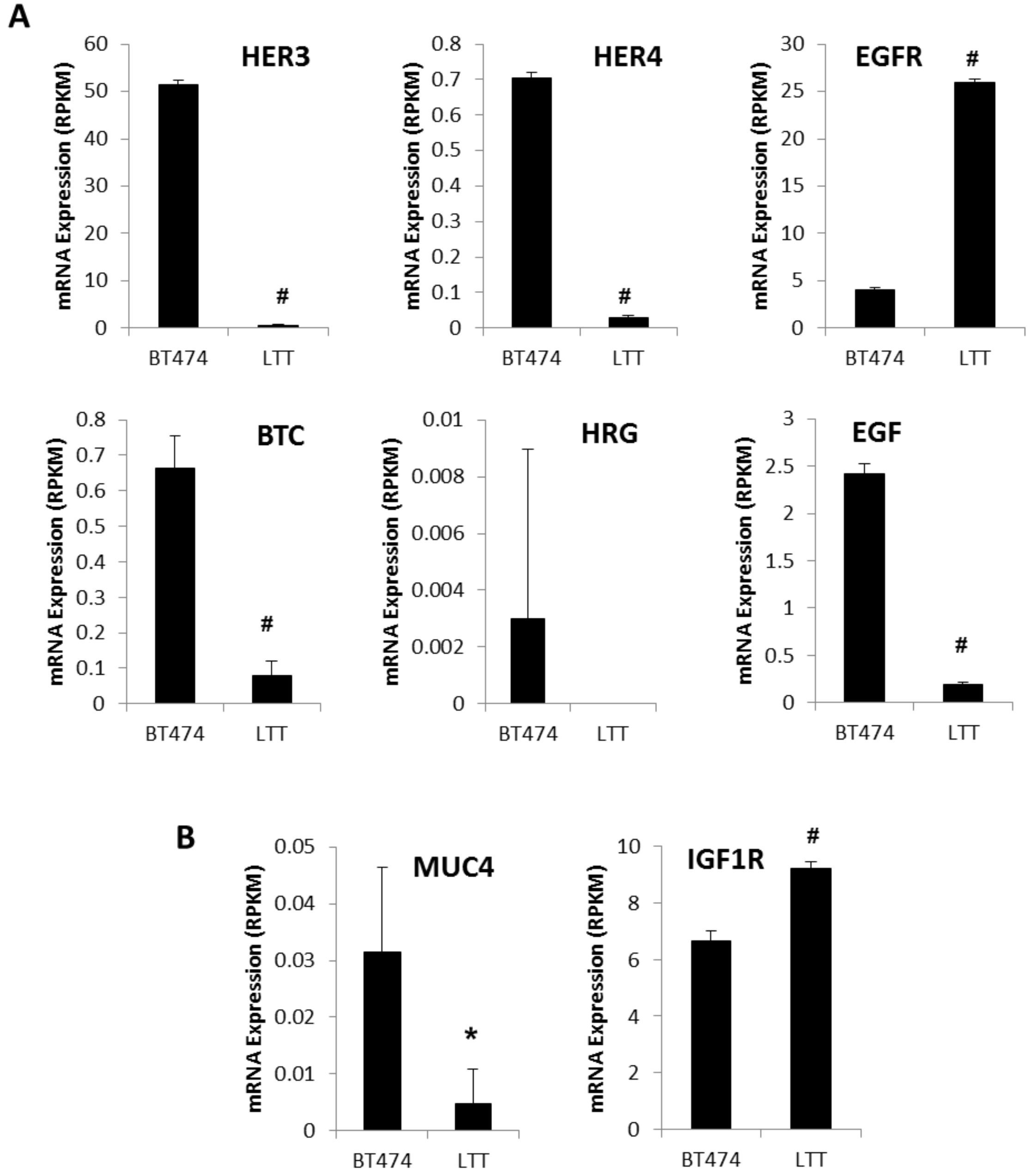

Figure S1. Generation of trastuzumab resistance from BT474 with PTEN deletion reduces ERBB family receptors and ligands with modest change in classical mechanisms associated with resistance. Normalized mRNA expression (reads/kilobase/million reads) of (A) ERBB family members HER3, HER4, and EGFR with corresponding major ligands and (B) genes previously associated with antigen masking on HER2 and resistance induced signaling crosstalk, MUC4 and IGF1R respectively. N=4. Data shown as average ± SD. * $p \leq 0.05$, # $p \leq 0.01$

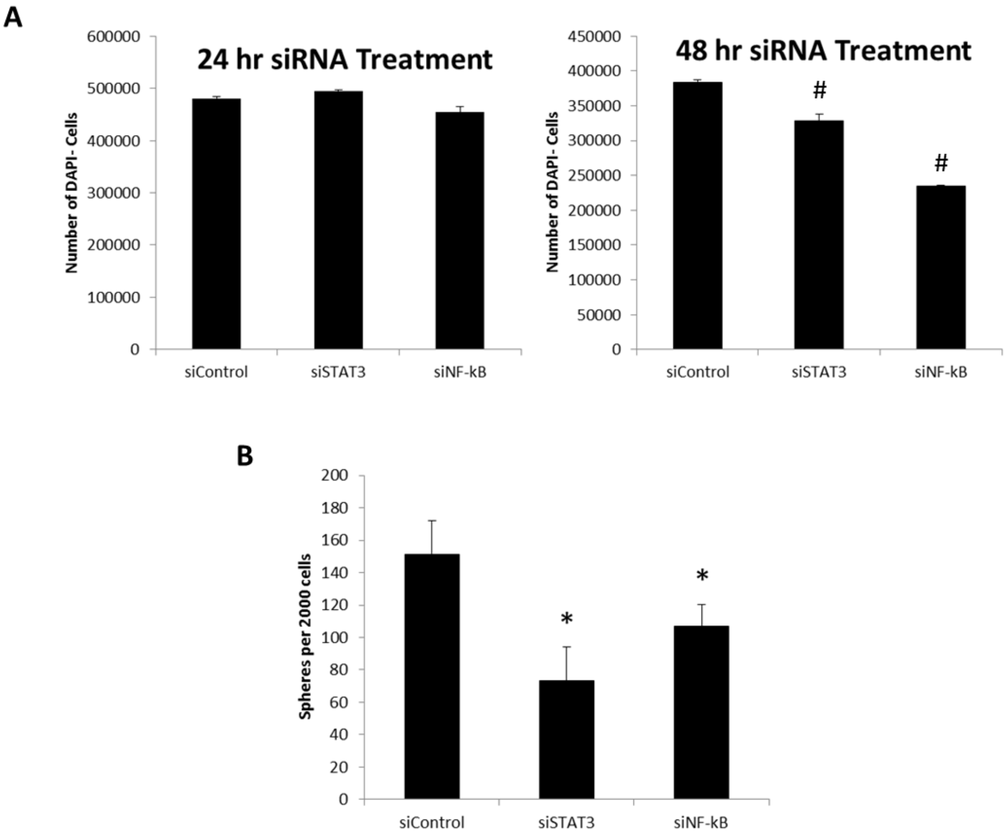

Figure S2. NF-kB regulates bulk cell line proliferation and cancer stem cells in trastuzumab resistant breast cancer with PTEN inactivation. (A) Flow cytometry analysis of total DAPI negative BT474 PTEN- LTT cells following siRNA knockdown of untargeted control, STAT3, or the p65 subunit of NF-κB 24 and 48 hours post transfection. N=3. (B) Number of mammospheres formed from BT474 PTEN- LTT cells following 7 day culture in serum free, nonadherent, conditions after siRNA transfection. N=3. Data shown as average ± SD. * $p \leq 0.05$, # $p \leq 0.01$
